# Factors Associated with Change in Sexual Transmission Risk Behavior over 3 Years among HIV-Infected Patients in Tanzania

**DOI:** 10.1371/journal.pone.0082974

**Published:** 2013-12-18

**Authors:** Brian W. Pence, Kathryn Whetten, Kristen G. Shirey, Jia Yao, Nathan M. Thielman, Rachel Whetten, Dafrosa Itemba, Venance Maro

**Affiliations:** 1 Department of Epidemiology, The University of North Carolina at Chapel Hill, Chapel Hill, North Carolina, United States of America; 2 Center for Health Policy & Inequalities Research, Duke Global Health Institute, Sanford School of Public Policy, Duke University, Durham, North Carolina, United States of America; 3 Department of Psychiatry and Behavioral Sciences, School of Medicine, Duke Global Health Institute, Duke University, Durham, North Carolina, United States of America; 4 Division of Infectious Diseases, Department of Medicine, Duke Global Health Institute, Duke University, Durham, North Carolina, United States of America; 5 Tanzania Women Research Foundation, Moshi, Tanzania; 6 Kilimanjaro Christian Medical Center, Moshi, Tanzania; University of Cape Town, South Africa

## Abstract

**Background:**

The reduction of HIV transmission risk behaviors among those infected with HIV remains a major global health priority. Psychosocial characteristics have proven to be important correlates of sexual transmission risk behaviors in high-income countries, but little attention has focused on the influence of psychosocial and psychological factors on sexual transmission risk behaviors in African cohorts.

**Methodology and Principal Findings:**

The CHAT Study enrolled a representative sample of 499 HIV-infected patients in established HIV care and 267 newly diagnosed HIV-infected individuals from the Kilimanjaro Region of Tanzania. Participants completed in-person interviews every 6 months for 3 years. Using logistic random effects models to account for repeated observations, we assessed sociodemographic, physical health, and psychosocial predictors of self-reported unprotected sexual intercourse. Among established patients, the proportion reporting any recent unprotected sex was stable, ranging between 6–13% over 3 years. Among newly diagnosed patients, the proportion reporting any unprotected sex dropped from 43% at baseline to 11–21% at 6–36 months. In multivariable models, higher odds of reported unprotected sex was associated with female gender, younger age, being married, better physical health, and greater post-traumatic stress symptoms. In addition, within-individual changes in post-traumatic stress over time coincided with increases in unprotected sex.

**Conclusions and Significance:**

Changes in post-traumatic stress symptomatology were associated with changes in sexual transmission risk behaviors in this sample of HIV-infected adults in Tanzania, suggesting the importance of investing in appropriate mental health screening and intervention services for HIV-infected patients, both to improve mental health and to support secondary prevention efforts.

## Introduction

The reduction of HIV transmission risk behaviors among those infected with HIV, or secondary HIV prevention, remains a major global health priority. While large investments have been made over the past decade to expand access to HIV treatment and medications,[1] and new evidence demonstrates that treatment will play a critical role in stemming new infections,[2] treatment and prevention remain inextricably linked in global efforts to combat the HIV epidemic.[3]

Among the predictors of sexual transmission risk behaviors, psychosocial characteristics such as depression, substance use, stressful and traumatic experiences, and post-traumatic stress have been identified in high-income countries as playing important roles,[4]-[10] leading to efforts to reduce sexual transmission through effective mental health interventions for HIV-infected individuals.[11]–[13] Although research in low- and middle-income countries have documented high prevalence rates of depression, trauma history, and other mental health concerns among HIV-infected individuals,[14]–[16] little attention has focused on the influence of psychosocial and psychological factors on HIV sexual transmission risk behaviors in African cohorts.

Accordingly, in the present paper we investigate predictors of sexual risk behaviors over 3 years of longitudinal follow-up among a cohort of over 750 newly diagnosed and established HIV-infected patients in Tanzania, with a particular emphasis on the association of psychological characteristics with sexual risk.

## Methods

### Study population and protocol

The Coping with HIV/AIDS in Tanzania (CHAT) Study is a longitudinal observational cohort study designed to define the determinants of HIV-related behaviors and health outcomes among Tanzanian adults in the Kilimanjaro Region. The CHAT Study includes 5 distinct cohorts: representative samples of HIV-infected patients engaged in routine clinical care at the regional tertiary referral hospital (n = 228) and largest public hospital (n = 271); consecutively recruited individuals newly testing HIV-seropositive at voluntary counseling and testing (VCT) centers (n = 267); individuals testing HIV-seronegative at the same VCT centers (n = 182); and a random sample of community-dwelling adults (n = 249). Study participants were recruited in 2008–2009 and completed in-person interviews in Swahili with trained local interviewers every six months through 2013. The study protocol has been described in greater detail previously.[17]

The present analysis focuses on predictors of self-reported sexual transmission risk behaviors, over the first three years of follow-up, among HIV-infected CHAT participants, i.e. those recruited from the two hospitals (“established HIV patients”) and those newly diagnosed at VCT centers (“newly diagnosed HIV patients”).

### Ethical approval

This study and all study activities were specifically approved by the Kilimanjaro Christian Medical Center Institutional Review Board in Tanzania and the Duke University Health System Institutional Review Board in the United States, and written informed consent was obtained from all participants.

### Measures

*Sexual transmission risk behaviors* were assessed via self-report during each in-person interview (every 6 months). Participants were asked to report the number of sexual partners in the past 6 months. Separately for primary and non-primary partners, participants were asked to report the total number of acts of anal or vaginal intercourse in the past 6 months and the number of those acts in which a condom was used. Measures taken to enhance the accuracy of these self-report items included anchoring (repeatedly orienting the participant to a date or participant-identified event six months ago), asking separately about primary vs. non-primary partners, and interviewer prompts to assist respondents, if necessary, in estimating frequency of intercourse by day, week, or month. The percentage of sexual acts that involved a condom was then calculated and reported back to the participant for confirmation or correction. Participants were also asked whether they had used a condom the last time they had sex with a primary and a non-primary partner, and whether they had exchanged (given or received) sex for gifts, money, shelter, food, or other benefit in the past 6 months (transactional sex).

Participants self-reported *sociodemographic information* at baseline including age, gender, marital status, highest level of completed education, religion, household assets, and tribe. *Overall health-related physical functioning* was assessed every 6 months with the Short Form (SF)-8, a validated shortened version of the extensively used SF-36.[18], [19] The Physical Composite Score (PCS) was computed according to standard methodology; this score is a weighted average of the SF-8 items with heaviest weights given to questions focusing on overall perception of physical health, healthy physical functioning (e.g., degree to which health interferes with walking and lifting), functioning without bodily pain, and healthy role functioning (e.g., extent to which health limits work and activities). The score can range from 0–100, with higher scores indicating better health-related physical functioning and 10 units representing one standard deviation in the US normative population.[20]

*Depressive severity* was assessed every 6 months with the Patient Health Questionnaire-9 (PHQ-9), a widely used depression case identification tool which has been validated in African populations[21], [22] and has a possible range of 0–27 with higher scores indicating greater depressive severity.[23], [24] *Post-traumatic stress (PTS) symptom severity* was assessed every 6 months with the PTSD Symptoms Checklist (PCL) based on DSM-IV criteria that include re-experiencing a traumatic event, numbing/avoiding, and hyperarousal symptoms. This scale has strong reported reliability, and correlates highly with a clinician-administered PTSD measure.[25], [26]

*The number of categories of lifetime potentially traumatic events*, adapted from prior research, included sexual abuse, severe physical trauma, childhood physical and emotional neglect, and other potentially traumatic experiences.[27]–[30] Sexual abuse was defined to include sexual experiences (e.g., touching, intercourse) where force or threat of force was used; however, in children (before the age of puberty) the threat of force or harm was implied by a 5-year age differential between the victim and perpetrator. Physical abuse was defined as incidents separate from sexual abuse that were perceived to be life threatening (being physically attacked with the intent to kill or seriously injure), and other physical abuse (being beaten, hit, kicked, bit, or burned). Childhood physical and emotional neglect was measured with the Childhood Trauma Questionnaire and scored using the cutoffs suggested by Bernstein and Fink for moderate physical neglect (≥9) and moderate emotional neglect (≥12).[27] Other potential traumas before age 18 were parental alcohol/drug abuse, depression, suicide or attempted suicide; imprisonment of a parent; domestic violence in the home; being placed in reform school, prison or jail, or foster or adoptive care; death of an immediate family member; and having a life-threatening illness or injury not related to HIV. Lifetime potential traumas included murder or death by trauma of a close family member, death of a child, and death of a spouse/partner. Participants were assigned a score from 0 to 15, reflecting the number of types of potentially traumatic events experienced in their lifetime. This specification of the number of types of potentially traumatic events experienced has been used widely and has been associated with multiple negative health outcomes.[31]–[35]

*Recent stressful life events* were measured every 6 months with a modified version of the Life Events Survey (LES)[36], [37] to measure the occurrence of stressful events in the 6 months preceding the baseline interview. Only those events considered to be moderately to severely stressful based on previous studies with interviewer-based objectively rated stresses were included.[34], [38] Moderate stressors included experiences such as relationship difficulties; death or serious illness of a close friend or immediate family member; employment difficulties (e.g. loss of job); and non-HIV-related serious illnesses, injuries, and accidents. Severe stressors included divorce/separation, death or illness of a close friend or immediate family member, major financial problems (e.g., loss of home), and more than a week in prison.

### Statistical analyses

Distributions of variables are summarized with means and standard deviations or percentages. We defined the primary outcome variable as a dichotomous measure of any reported unprotected sexual intercourse with a primary or non-primary partner in the past 6 months. We used random effects logistic regression models with an unstructured correlation matrix to estimate the associations between any reported unprotected sex and predictor variables while accounting for correlations between repeated observations on the same individual. In this model, we included outcomes data from rounds 2–7 in order to focus on the period when all participants were aware they were HIV-infected. The model included random intercepts for each participant to account for the natural heterogeneity among participants. The appropriateness of modeling continuous variables using simple linear terms was confirmed through visual examination of locally weighted scatterplot smoothing (lowess) graphs and comparison of linear to more flexible (e.g. quadratic) modeling choices. The potential for model instability due to collinearity was evaluated by assessing whether standard errors for coefficients changed substantially as potentially correlated variables were added to the model.

The multivariable model included predictors that were of interest a priori, with a particular focus on the association between time-varying psychosocial characteristics and reported unprotected sex. Each time-varying predictor (physical health, PTS symptoms, and stressful events) is represented in the models by two terms.[39] The first term (constant across all time points) was calculated as the individual's mean score on that item across all time points, and the second (varying across time points) was calculated as the deviation (difference) between an individual's overall mean and her score at each particular time point. Coefficients on the first set of terms represent between-person associations (e.g., do people with higher average PTS symptom severity engage in more sexual risk behaviors than people with lower severity?) while coefficients on the second set of terms represent within-person associations, in effect allowing each person to serve as her own control (e.g., if at a given interview an individual reports higher PTS symptom severity than her norm, does she also report more sexual risk behaviors?). This decomposition is important since between-person and within-person effects are not always of the same magnitude or even in the same direction.[39]

Two potential confounders, alcohol use and fertility intentions, were only available from a subset of time points (alcohol use: rounds 4–7; fertility intentions: rounds 3 and 7). Therefore these variables were not included in the primary model, but the potential for uncontrolled confounding was assessed by comparing models with and without these variables in the sample of observations for which measures were available. Effect modification by gender and marital status was assessed via likelihood ratio tests comparing models with and without interaction terms between these variables and key psychosocial variables of interest, with a likelihood ratio P value <0.05 considered indicative of effect measure modification.

All analyses were conducted using STATA version 11 (Stata Corporation, College Station, TX).

## Results

### Sample description

Of 766 HIV-infected participants enrolled, 755 (493 established, 282 newly diagnosed) provided information on sexual behaviors and are included in the analyses (Table 1). Follow-up rates ranged from 88–92% over 36 months for the established patients and from 69–77% for the newly diagnosed patients. The mean (SD) age of the sample was 40.3 (9.1) years. Approximately two-thirds of participants were female. Approximately 40% of participants were currently married or cohabitating with a long-term partner at baseline, with those widowed and those divorced representing approximately 22% each, and those never married representing 16%. Approximately 17% of participants had received education beyond primary school. Participants had experienced a mean (SD) of 2.3 (1.7) traumatic events in their lifetime and 3.1 (2.2) stressful events in the past 6 months.

**Table 1:** Description of Sample.

Characteristic	Range	Mean	SD	n	%
Total sample				755	100.0
Female gender				515	68.2
Age (years)	18–65	40.3	9.1		
Marital status					
Married or cohabitating				297	39.4
Widowed				170	22.6
Divorced				166	22.0
Never married				121	16.1
Education beyond primary school				133	17.6
Established (vs. newly diagnosed) HIV patient				493	65.3
Physical health (SF-8)	15–66	47.5	10.1		
Depressive severity (PHQ-9)	0–27	4.5	6		
PTS severity (PCL)	0–42	8.7	7		
Recent stressful life events	0–13	3.1	2.2		
Number of lifetime traumatic experiences	0–13	2.3	1.7		
					

### Sexual behaviors

At baseline, a majority of unmarried participants reported no sexual partners in the past 6 months, and a majority of married or cohabitating participants reported 1 sexual partner; between 3–14% of participants reported 2 or more sexual partners in the past 6 months (Table 2). Among newly diagnosed patients, 28% of unmarried and 65% of married respondents reported any unprotected sex in the past 6 months; among established HIV patients, unprotected sex was reported by 5% and 18% of married and unmarried participants respectively. The proportion reporting any unprotected sex with a non-primary partner was lower among established patients (1–2%) compared to newly diagnosed patients (5–9%). The proportion of respondents reporting any transactional sex was also lower among established patients (2–4%) compared to newly diagnosed patients (8–14%).

**Table 2:** Self-reported sexual risk behaviors at baseline among CHAT sample.

	Newly diagnosed, unmarried		Newly diagnosed, married		Established, unmarried		Established, married	
Behavior	n	%	n	%	n	%	n	%
n	155	100.0	107	100.0	302	100.0	190	100.0
Number of sexual partners, past 6 months								
0	91	58.7	13	12.3	241	80.3	37	19.6
1	43	27.7	87	82.1	51	17.0	144	76.2
2 or more	21	13.6	6	5.6	8	2.7	8	4.2
Mean (SD)	0.7	1.3	0.9	0.5	0.2	0.6	0.9	0.5
Any recent unprotected sex	44	28.4	69	64.5	16	5.3	35	18.4
Any recent unprotected sex with a non-primary partner	16	10.3	8	7.5	3	1.0	3	1.6
Last sex with a non-primary partner was unprotected	14	9.0	5	4.7	3	1.0	3	1.6
Any exchange for sex in past 6 months	22	14.2	9	8.4	12	4.0	3	1.6
								

**Figure 1:**
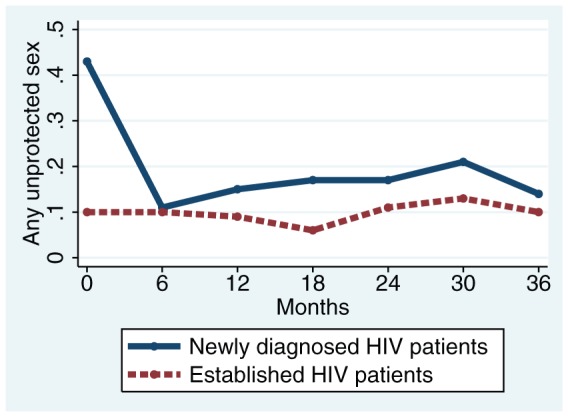
Trajectory of unprotected sexual intercourse over 3 years among HIV patients in Tanzania.

Over time, the proportion of established patients reporting any recent unprotected sex remained fairly stable, ranging between 6% and 13% over 3 years (Figure 1). Among newly diagnosed patients, the proportion reporting any unprotected sex dropped from 43% at baseline (corresponding to the 6 months prior to testing positive for HIV) to 11% at 6 months (corresponding to the 6 months after being diagnosed), and thereafter remained fairly stable between 11–21% through 3 years.

### Predictors of reported sexual risk

In multivariable logistic regression random effects models controlling for correlation between repeated measures on each individual (Table 3), female gender was associated with higher odds of reporting unprotected sex (OR = 1.62, 95% CI = 0.99–2.67) and increasing age was associated with lower odds (OR per year = 0.97, 0.95–0.99; meaning the predicted change in odds for a 10–year increase in age would be 0.73, 0.57–0.94). Compared to those currently married or cohabitating, those widowed or divorced (OR = 0.26, 0.18–0.39) and those never married (OR = 0.13, 0.06–0.25) were less likely to report unprotected sex. Established HIV patients tended to be less likely to report unprotected sex than newly diagnosed patients (OR = 0.59, 0.33–1.06), even after omitting the baseline data corresponding to the period before the newly diagnosed had tested positive for HIV. Antiretroviral therapy was associated with lower odds of reported unprotected sex (OR = 0.43, 0.26–0.70).

**Table 3:** Multivariable model of predictors of unprotected sexual intercourse.

Predictor	Risk Ratio	95% CI
Female gender	1.62	(0.99, 2.67)
Age (per year)	0.97	(0.95, 0.99)
Marital status		
Married or cohabitating	1.	ref
Widowed or divorced	0.26	(0.18, 0.39)
Never married	0.13	(0.06, 0.25)
Education beyond primary school	1.49	(0.84, 2.66)
Physical health (SF-8)		
Mean	1.10	(1.01, 1.20)
Deviation	1.01	(0.98, 1.03)
Depressive severity		
Mean	0.87	(0.74, 1.04)
Deviation	1.01	(0.95, 1.07)
PTS severity		
Mean	1.13	(1.07, 1.20)
Deviation	1.02	(1.00, 1.05)
Recent stressful events		
Mean	1.06	(0.88, 1.28)
Deviation	1.01	(0.94, 1.08)
Number of traumatic life events	1.14	(0.95, 1.36)
Established (vs. newly diagnosed) HIV patient	0.59	(0.33, 1.06)
On antiretroviral medication	0.43	(0.26, 0.70)
		
*Random effects: SD (95% CI)*		
Participant random intercepts	1.70	(1.45, 1.99)
		
-2LL Wald chi-square (df), P value		114.8 (16), <0.001
		

When considering time-varying covariates, those with better mean physical health scores were more likely to report unprotected sex (OR = 1.10, 1.01–1.20 per 1 point on the SF-8 physical composite index), but within-person changes in physical health over time were not associated with changes in sexual risk (OR = 1.01, 0.98–1.03). Those with higher mean post-traumatic stress symptom severity scores were also more likely to report unprotected sex (OR = 1.13, 1.07–1.20 per 1 point on the PCL). Moreover, within-person changes in PTS symptom severity over time were also associated with changes in sexual risk (OR = 1.02, 1.00–1.05 per 1 point increase), meaning that if an individual scored 5 points above her norm in PTS symptom severity at a given interview, she had a 13% increased odds of reporting unprotected sex in the 6 months before that interview. Depression and recent stressful events were not associated with overall sexual risk or within-person changes in sexual risk over time.

Likelihood ratio tests did not indicate presence of effect measure modification of the above associations by gender or marital status. Comparison of models including and excluding fertility intentions and alcohol use in the subset of observations with information on those variables indicated that neither variable was likely to represent substantial uncontrolled confounding in the final full sample model. Model diagnostics indicated that while several psychosocial predictor variables included in the final model were moderately correlated (ρ 0.4–0.7), collinearity did not affect model stability (as measured by changes in standard errors of coefficients as variables were sequentially added) or substantive conclusions.

## Discussion

In this study, HIV-infected participants reported stable rates of unprotected sexual intercourse over 3 years of follow-up. While nearly half of newly diagnosed patients reported unprotected sex in the 6 months before testing positive for HIV, the proportion dropped to approximately 10–20 percent after diagnosis. Patients with established HIV infection, meanwhile, reported even lower rates. Unprotected sex with a non-primary partner or by unmarried participants was rarely reported, as was transactional sex. Even within marriage, unprotected sex was unusual among patients with established HIV infection. For comparison, in studies of male circumcision as an HIV prevention strategy in Uganda, 85–86% of participants reported some unprotected sex at baseline.[40], [41] While any unprotected sex presents some transmission risk, the low frequency of multiple partners, unprotected sex, and transactional sex suggests largely appropriate secondary prevention practices, especially among those with established HIV infection, and may reflect the impact of safer sex messages routinely delivered in the region in HIV clinical care and at the time of HIV testing.

We found that individuals with higher levels of post-traumatic stress symptomatology were more likely to report unprotected sex. Further, for a given individual, an increase in PTS symptoms during longitudinal follow-up relative to the individual's overall mean was linked to an increase in the likelihood of reporting unprotected sex. These findings are consistent with other reports that have linked sexual risk behaviors with mental health symptomatology and exposure to traumatic experiences.[4], [42], [43] While caution must be used in assigning causal interpretations to observational data, the contemporaneous changes in PTS symptoms and reported sexual risk during longitudinal follow-up support an effect of trauma-related mental health symptomatology on risk behaviors in this Tanzanian population of newly diagnosed and established HIV patients.

The finding in this sample that PTS symptom severity was associated with sexual risk, while number of lifetime traumatic experiences was not, is consistent with literature demonstrating marked changes in the brain associated with PTSD. For example, MRI studies of brain volume have found smaller hippocampal volumes, among other structural brain differences, in trauma-exposed individuals with PTSD compared to trauma-exposed individuals without PTSD.[44] Given the critical role the hippocampus plays in learning and memory, it is plausible that those with PTSD may have different risk behaviors than those exposed to trauma but without current PTSD symptoms.

In considering the absence of an association between traumatic life events and sexual risk in the present analysis, it is worth noting the very high prevalence of exposure to traumatic life events in the CHAT sample. Nearly all participants in the present analysis (88% of established and 85% of newly diagnosed HIV patients) had experienced at least one traumatic life event, with a mean of 2.3 lifetime experiences. Thus the coefficient on the variable representing lifetime traumatic experiences, modeled continuously, represents less a contrast between those with any vs. no traumatic experiences but rather a contrast between those with more vs. fewer experiences.

We also identified gender differences in reported unprotected sex, with women more likely to report unprotected sex than men. This may reflect gender differences in ability to negotiate condom use, as has been described in Uganda.[45] It may also reflect differences in disclosure of HIV status: due to fears of being ostracized, women may be less likely to have disclosed their HIV status to their partner, and may be reluctant to suggest condom use without disclosing their status.[46]–[48]

Notable strengths of this study include the large sample size, systematic sampling approach, inclusion of both established and newly diagnosed patients, longitudinal design, and analytic approach that specifically identifies predictors for which changes over time are associated with changes in reported sexual risk. One limitation of this study is that sexual risk behaviors were collected via participant self-report, raising the possibility of social desirability bias. Interviewers were carefully trained not to imply any judgment in administering these questions, and respondents were assured that all information would be kept confidential. Also, these data do not suggest increasing reports of sexual risk over time, which might be expected if participants were more willing to disclose risk behaviors as they developed stronger rapport with interviewers over time. An additional limitation is that the serostatus of the sexual partners was not collected, thus we cannot distinguish unprotected sex with a seronegative partner from unprotected sex between two HIV-infected individuals. However, it is important to note that even between two HIV-infected individuals, use of a condom is recommended to avoid the risk of super-infection with a heterologous HIV strain.

As described in detail elsewhere, recruitment strategies for the CHAT cohort were carefully designed to yield a representative sample of HIV-infected patients in established care and individuals newly diagnosed with HIV in the study area.[17] While retention among established patients was nearly 90% through 3 years, attrition was higher among the newly diagnosed participants, with approximately 30% either dying or being lost to follow-up by 3 years. Attrition by 3 years was not associated with either psychosocial factors or reported sexual risk at enrollment, suggesting that it is unlikely to have affected the primary results from this analysis.

The present sample was similar in physical health measures to some other studies of HIV-infected individuals with comparable measures. Compared to the SF-8 physical composite score mean of 47.5 in the present sample, Fox et al. reported a mean of 48.2 in an oral health study of HIV-infected individuals throughout the US,[49] while the CHASE study of HIV-infected individuals in the southeastern US had a mean SF-36 PCS score of 45.7.[34] Mental health symptomatology scores were also generally comparable if somewhat lower (healthier) in the CHAT sample than in some comparison samples, although there are relatively few examples from sub-Saharan Africa. The mean number of lifetime traumatic experiences in the CHASE sample was 3.1 compared to 2.3 in the present sample, and the mean PTSD score was 13.9 compared to 8.7 in the present sample.[50] Among a sample of HIV-infected patients in Kenya, a mean depressive severity score of 7.2 on the PHQ-9 was reported, compared to a mean of 4.5 in the present sample.[22]

Other studies have also documented associations between mental health symptomatology and sexual risk behaviors among HIV-infected individuals. While the majority of such research has been conducted in high-income countries,[4]–[10] emerging evidence supports similar relationships in low- and middle-income countries in sub-Saharan Africa and elsewhere.[16], [51] Post-traumatic stress symptomatology is characterized by distressing and intrusive thoughts, with which the individual may try to cope through dissociation, substance use, or other strategies which can affect the ability to assess and manage risk.[52]–[54] Hyperarousal, another core symptom of post-traumatic stress, can also affect the individual's ability to accurately assess risk.[52], [55] Interventions to address the sequelae and symptomatology of stressful events and traumatic experiences have shown promise in reducing sexual transmission risk behaviors among HIV-infected individuals with histories of trauma;[11], [12] while such interventions have primarily been developed in high-income countries, increasing emphasis is being placed on the expansion of evidence-based mental health services and the integration of quality mental health care with other medical services for HIV-infected patients in low-income countries.[56], [57]

Every HIV sexual transmission event involves one HIV-infected and one HIV-uninfected partner, inextricably linking primary and secondary HIV prevention efforts. The present study highlights the longitudinal association of changes in post-traumatic stress symptomatology and sexual transmission risk behaviors among HIV-infected adults in Tanzania, suggesting the importance of investing in appropriate mental health screening and intervention services for HIV-infected patients, both to improve mental health and to support sexual transmission prevention efforts.
